# Adverse Events in Patients With Rheumatoid Arthritis and Psoriatic Arthritis Receiving Long-Term Biological Agents in a Real-Life Setting

**DOI:** 10.3389/fphar.2019.00965

**Published:** 2019-09-11

**Authors:** Mayara Costa de Camargo, Bruna Cipriano Almeida Barros, Izabela Fulone, Marcus Tolentino Silva, Miriam Sanches do Nascimento Silveira, Iara Alves de Camargo, Silvio Barberato-Filho, Fernando de Sá Del Fiol, Luciane Cruz Lopes

**Affiliations:** ^1^Pharmaceutical Sciences Graduate Program, University of Sorocaba (UNISO), Sorocaba, Brazil; ^2^Hematology Graduate Program, Federal University of São Paulo (UNIFESP), São Paulo, Brazil

**Keywords:** adverse reaction, biologic agents, psoriatic arthritis, rheumatoid arthritis, safety

## Abstract

**Background:** Biological agents used for the treatment of psoriatic arthritis (PsA) and rheumatoid arthritis (RA) are associated with serious adverse effects (SAEs). Although several biologics have demonstrated good efficacy and tolerability in short-term trials, treatment guidelines recommend them as third line therapies due to a relative lack of long-term safety data.

**Objective:** To determine the frequency and severity of adverse effects associated with the long-term use of biologics in the treatment of PsA and RA, and possible risk factors for such events in a real-life setting.

**Methods:** We conducted a longitudinal study in PsA and RA patients only taking long-term biological agents from 2003 to 2011. Sources of information included dispensing pharmacy data and interviews with patients. Research staff conducted telephone interviews with patients inquiring about any apparent medication-related adverse drug reactions (ADRs) or SAEs. ADR/SAE’s data was based on pharmacy reports. We conducted a multivariate analysis to identify the factors associated with the risk of ADRs.

**Results:** Of the 305 patients identified, we interviewed 268 patients. Most of these were taking adalimumab 127 (47.4%), 52 (19.4%) etanercept, 42 (15.7%) infliximab, 25 (9.3%) rituximab, 10 (3.7%) abatacept, 9 (3.4%) efalizumab, and 3 (1.1%) tocilizumab. Of the 268 patients, 116 (43.3%) experienced one or more adverse events related to biological agents with 1.6 events per patient, and of these 29 (25%) experienced one or more SAEs, with majority subjected to hospitalizations. The most frequently reported ADRs were administration site reactions as observed in 73 patients (27.2%), infections in 30 patients (11.2%), effects on nervous system in 22 patients (8.2%), and 15 (5.6%) patients withdrew due to ADRs. The use of rituximab was related with less risk of ADR [PR 0.42, 95% CI 0.18–0.96; *p* = 0.04] than other agents. No other predisposing factors were associated with risk of ADR. The monitoring of patients (medical consultation and laboratory test) was only completed by 48 patients (30.4%).

**Conclusion:** These data showed the early biological experience in Brazil that were associated with ADRs, withdrawals due to ADRs and SAEs. The quantification of adverse effects (serious or nonserious) considering close monitoring and patients’ perceptions are increasingly important for future decision-making.

## Introduction

Biologic agents, introduced in the late 1990s, have improved the treatment outcomes of autoimmune disease, inflammatory disease, and tumour therapy (Chen et al., 2006; NICE, 2012; Coates et al., 2013). Additionally, the application of biological processes involving recombinant DNA technology, which allowed the production of proteins like cytokines and humanized antibodies, must be credited (Mazurek and Jahnz-Rozyk, 2012).

These drugs include tumour necrosis factor (TNF) inhibitors (e.g. adalimumab, certolizumab, etanercept, golimumab and infliximab), anti-CD28 agent (abatacept), anti-cytokine agents (anakinra and tocilizumab), anti-B-cell agent (rituximab), T-cell modulating agent (alefacept), and inhibitors of interleukin (IL)-12 and IL-23 (ustekinumab) (Rosman et al., 2013). Indications for use vary between the countries in which they have been approved for marketing.

The wide use of biological agents in modern medicine is a challenge for physicians and requires constant learning, with distinct knowledge and familiarity of the disease to be treated. Additionally, these biological agents are expensive and compel the physicians to consider the economic burden on patients. Biologic agents have been associated with high rates of total adverse events and withdrawals due to adverse events (Singh et al., 2011).

Tumor Necrosis Factor-alpha (TNFα) is essential for increasing phagocytic activity of macrophages and other killer cells; therefore, anti-TNFα medication can lead to common and opportunistic infections (Curtis et al., 2011). These include tuberculosis, atypical mycobacteriosis, listeriosis, histoplasmosis, aspergillosis, pneumocystis, and legionellosis (Murdaca et al., 2015).

There is increasing evidence of the paradoxical induction of autoimmune processes associated with biological agents (Lee and Kavanaugh, 2005; Katz and Zandman-Goddard, 2010; Karmacharya et al., 2015). Autoimmune diseases secondary to biological therapies comprise a variety of both, systemic illnesses including lupus, vasculitis, sarcoidosis, and antiphospholipid syndrome (Pichler, 2006). Biological agents have also been associated with organ-specific autoimmune processes including interstitial lung disease, uveitis, optic neuritis, peripheral neuropathies, multiple sclerosis, and autoimmune hepatitis (Hausmann et al., 2010).

The majority of adverse effects manifest between one month to one year after initiating the therapy with a biological agent; however, they may also manifest years after treatment suspension (Mazurek and Jahnz-Rozyk, 2012). Biological agents may also manifest adverse effects that are yet unknown, suggesting that monitoring of ongoing patients is essential (NICE, 2012; Silveira et al., 2014; Gonzalez-Alvaro et al., 2015; Deighton et al., 2009).

Patient reports are an important source of information on patient safety (Fowler et al., 2008; Kuzel et al., 2004) and are useful in evaluating the adverse events (Hibbard et al., 2005; Zhu et al., 2011). In Brazil, patients reported one or more adverse reactions associated with biological agents in 67% of the cases. These patients had no close clinical monitoring (Lopes et al., 2014; Camargo et al., 2016).

To further elucidate the adverse effects and predisposing factors associated with the use of biologics in clinical practice, we performed an observational study in patients with psoriatic arthritis (PsA) and/or rheumatoid arthritis (RA) who had been using the same biological agent for at least six months. The main objective was to evaluated the medium- and long-term safety of biologics in patients from a middle-income country.

## Methods

### Design and Setting

We utilized a retrospective longitudinal design to investigate the adverse drug reactions (ADRs) (Gonzalez-Alvaro et al., 2015) occurring in patients using long-term biologics (abatacept, adalimumab, efalizumab, etanercept, infliximab, rituximab, and tocilizumab) for treatment of PsA and RA in Brazil. The protocol was authorized by the Health State Department and approved by the Ethics Committee for Clinical Research of University of Sorocaba (August 17, 2009; protocol number 011/2009). Each patient provided an informed consent.

### Definitions

We defined ADR as “a response to a medicine or medicinal product that is noxious and unintended, and which occurs in doses normally used in humans for the prophylaxis, diagnosis, or therapy of disease or for the modification of physiological function” (WHO, 2002). Therefore, we excluded events that resulted from drug errors, therapeutic failures, intentional or accidental poisoning, and drug abuse.

A serious adverse event (SAEs) was defined under the same code as ‘any untoward medical occurrence that at any dose results in death; is life-threatening; requires inpatient hospitalization or prolongation of existing hospitalization; creates persistent or significant disability/incapacity, or a congenital anomaly/birth defect’ (International Council on Harmonisation, 1994).

### Eligibility Criteria

Eligible patients were those who underwent treatment with biologics for PsA/RA for at least 6 months, during 2003–2011.

### Identification of Patients and Collection of Patient Data

To identify eligible patients, two researchers abstracted data from all the dispensing orders from the database of the government (CODES-SP). The patients with PsA were identified by ICD code M07, and those with RA were identified by ICD code M05. Patient details such as name, address, telephone number, gender, age, healthcare provider, type of biologic dispensed, and duration of treatment and diagnoses were collected.

We contacted these patients by telephone, and if they proved eligible and agreed to participate in the study, we conducted interviews by telephone using a questionnaire. The questionnaire included the following: name of the drug that patient was using for the treatment of PsA and RA; time of diagnosis of the disease; comorbidities; adverse drug reaction and whether it led to discontinuing medications; and whether patients was informed about the risk of taking such drugs.

Interviews were conducted by telephone using computer-assisted telephonic interview technology with a microcomputer handset with headphones. This system allows recording and monitoring of the conversation. Research staff working in pairs independently recorded data from the interviews, with discrepancies (if any) resolved by the principal investigator (LCL). This interview approach was developed by local dermatologists and rheumatologists in accordance with the recommendations of Brazilian and others important guidelines. Each interviewer (pharmacists) received training on use of language, related to each question in the interview schedule.

In order to dispense the biologics drugs in the pharmacy of the government, all pharmacists monitoring the patients with a formal structured checklist for ADR validated by rheumatologist and dermatologists.

We crosschecked patients’ reports with data obtained from pharmacy records and from the database of the government. If discrepancies were found between sources of information, we considered the information from the pharmacy records as definitive. Definitive information about the name of the biologic and the duration of its use was obtained from the pharmacy, and definitive information regarding the time of diagnosis, use of previous medicines, and laboratory results were obtained from the patient.

This study is a part of a protocol published elsewhere (Camargo et al., 2016; Silveira et al., 2014).

### ADR Reporting

We collected information regarding the reported ADRs to understand their onset, nature of the reaction (system or organ affected), causality (Naranjo et al., 1981), and severity (ICH, 1994). The Naranjo algorithm (Naranjo et al., 1981) provided guidance for establishing causality and ADRs were ranked in three categories (definite, probable, and possible). The ADRs ranked as “definite” and “probable” were classified as likely caused by the biological agent. Pairs of reviewers (SBF, BCAB, FSDF, MCC) independently classified potential ADRs as “present” or “absent,” and if present, classified them according to the causality classification. In case of disagreement a third reviewer (LL) provided adjudication.

### Predisposing Factors

We considered the following variables as possible predisposing factors for ADRs. For each factor, we priori postulated the direction of the possible effects: i) **age** (older [≥60 years] versus younger [19–59 years], with a higher risk in older) (Girolomoni et al., 2012), ii) **presence of comorbidities** (none versus one or more, with a higher risk in one or more illnesses) (Mazurek and Jahnz-Rozyk, 2012), iii) **diagnosis of more than one immunosuppressive disease** (only one versus more than one, with a higher risk for diagnostic with one or more) (Coates et al., 2013), iv) **concomitant use of other medications** (none versus one versus two or more, with a higher risk with more than one medication) (Girolomoni et al., 2012), v) **concomitant use of disease modifying anti rheumatic drugs—DMARD** (none versus one or more, with a higher risk with more than one DMARD) (Mazurek and Jahnz-Rozyk, 2012), vi) **physician provided warnings regarding risk of medication** (higher risk when warnings were provided) (Girolomoni et al., 2012), vii) **health insurance** (private versus public health insurance, with a higher risk for private insurance) (Mariette et al., 2011), viii) **biological agent**, iv) **duration of use of biologics** (6 to 12 months versus 13 months or more).

### Follow-Up and Clinical Monitoring

Clinical monitoring was done only in patients who were taking biologics during the interviews. We referred the guidelines of a few countries, such as Brazil (BRAZIL, 2014; BRAZIL, 2006), England (Smith et al., 2009), Canada (Bykerk et al., 2012), Germany (Wollenhaupt et al., 2013), European League against Rheumatism – EULAR (Gossec et al., 2016), Group for Research and Assessment of Psoriasis and Psoriatic Arthritis – GRAPPA (Coates et al., 2016) and took into account the common recommendations such as medical consults, laboratory blood examination, and radiograph.

As there is lack of consensus regarding the best interval for patient monitoring, we adopted the recommendations present in the Brazilian clinical protocols of the time, which considers as follows: **i) number of consults** (at least two annual medical consults); **ii) laboratory blood examination** (complete Blood Count, liver function tests and C-Reactive Protein test performed once a year) and **iii) radiography** (at least once a year). The IGRA sign test (interferon gamma release assay) was only introduced in public health service in 2014.

Owing to their high cost, biologics are only provided by government pharmacies, and therefore the follow-up was in accordance with the Brazilian official protocol. At the time of the study, annual radiography was considered as a test for monitoring tuberculosis progression, a condition common in Brazil, given the high number of patients acquired immunodeficiency syndrome (AIDS) in the general population.

### Statistical Analysis

Initially, we obtained the descriptive statistics of the variables studied through frequencies. Later, the variables were stratified by patients, with and without ADRs and SAEs. We calculated the prevalence ratio (PR) to detect factors associated with the risk of ADRs using a Poisson regression.

We used with bivariate analysis (unadjusted) as a first step, and then, the analysis was adjusted for age, presence of comorbidities and use of other concomitant drugs. A significance level of *p* < 0.05 and a confidence interval of 95% were adopted. All analyses were performed using STATA software.

## Results

**Figure 1:**
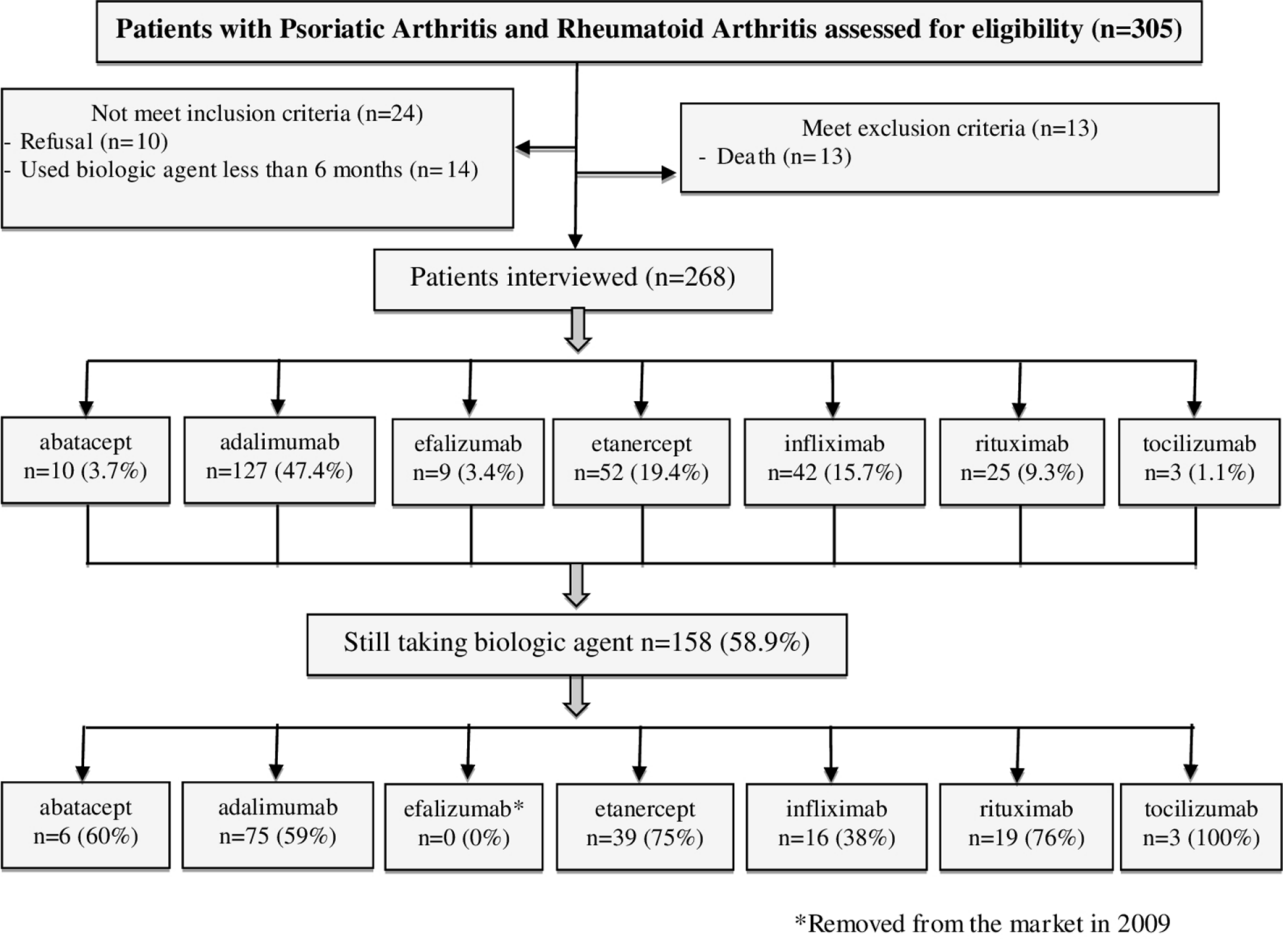
Flow diagram of the steps of the sample composition.

Of the 305 patients identified for using biologics for PsA or RA, 10 patients refused to participate, 13 were deceased and 14 used biological agent for less than 6 months. The resulting group of interviewees included, 268 plaintiffs of whom 158 (58.9%) were still using a biological agent at the time of the interview (**Figure 1**).

**Table 1** presents characteristics of the patients with PsA and RA. Most of the patients were female (73.1%), less than 60 years old (mean age 55.8 ± 13), with rheumatoid arthritis only (73.1%), with one or more comorbidity (51.5%), using the biologic for 13 to 36 months (mean duration 35.7 ± 20).

**Table 1:** Characteristics of the patients with psoriatic arthritis and rheumatoid arthritis.

Patients	n (%) 268 (100%)
**Sex**	
Female	196 (73.1)
Male	72 (26.9)
**Age**	
19–59	156 (58.2)
60 or more	112 (41.8)
mean ± sd	55.8 ± 13
**Diagnostic**	
Psoriatic arthritis only	63 (23.6)
Rheumatoid arthritis only	196 (73.1)
RA+PsA	9 (3.3)
Comorbidity*	
None	130 (48.5)
1 or more	138 (51.5)
Psoriasis	52 (37.7)
Cardiovascular	61(44.2)
Metabolic	34 (24.6)
Skeletal muscle	35 (25.4)
Others	28 (20.3)
**Duration use of biologic agents (months)**	
6 – 12	40 (14.9)
13 – 36	133 (49.6)
37 or more	95 (35.4)
mean ± sd	35.7 ± 20
**Health care**	
Private	56 (20.9)
Public	212 (79.1)
**Concomitant use of drugs with biologic agent**	
None	81 (30.2)
1	73 (27.2)
2 or more	114 (42.6)
**DMARD used with biologic agent**	
metrotexate	57 (21.3)
corticosteroids	24 (8.9)
Others	64 (23.9)
**Time since diagnostic of disease RA/PSAR**	
<1 years	5 (1.9)
1–3 years	9 (3.3)
3–5 years	31 (11.6)
>6 years	223 (83.2)

*Patients may have more than one comorbidity.

PsA, Psoriatic arthritis; RA, rheumatoid arthritis.

Patients with RA showed different comorbidities as compared to those with PsA. Approximately, 102 (52%) patients with RA showed comorbidities and 46 (45.1%) had cardiovascular diseases. Twenty-two (21.5%) patients had metabolic diseases (mainly diabetes and obesity), while 17 (16.6%) had muscle pain that rendered work disabilities. On the other hand, 16 (25%) patients with PsA showed comorbidities and 8 (50%) had cardiovascular disease, 6 (37.5%) had dermatological problems, 4 (25%) had inflammatory bowel disease, 3 (18.8%) had metabolic disease, 3 (18.8%) had musculoskeletal problems including osteoporosis and ophthalmic disorders was observed in 2 patients (12.5%).

**Table 2** depicts the characteristics of the adverse events in patients taking biological agents. Of the 268 patients, 116 (43.3%) experienced one or more adverse events related to the use of biological agents, at the rate of 1.6 events per patient. The most frequently [n = 73 (27.2%)] reported ADRs were administration site reactions (hypersensitivity reactions and cytokine-release syndrome), followed by incidences of respiratory and other types of infections [n = 30 (11.2%)] (opportunistic infections, urinary tract infections, skin infections, other systemic fungal infections and meninges infection)and effects on nervous system [n = 22 (8.2%)] (headaches and neuropathies). Of 116 patients with at least one ADR, 29 (25%) experienced SAE. The main causes for SAE included serious infections, malignancies and major cardiovascular events. There was no case of tuberculosis. We could not access the medical records to gain further details about these events. Eight patients required hospitalization, 6 had prolongation in existing hospitalization and 8 experienced life-threatening events.

**Table 2:** Characteristics of adverse events in patients taking biological agents to treat psoriatic arthritis and rheumatoid arthritis.

Variables	abatacept (n = 10)		adalimumab (n = 127)		efalizumab (n = 9)		etanercept (n = 52)		infliximab (n = 42)		rituximab (n = 25)		tocilizumab (n = 3)		TOTAL (n = 268)	
	Number of Patients with ADR	Number of Events	Number of Patients with ADR	Number of Events	Number of Patients with ADR	Number of Events	Number of Patients with ADR	Number of Events	Number of Patients with ADR	Number of Events	Number of Patients with ADR	Number of Events	Number of Patients with ADR	Number of Events	Number of Patients with ADR n (%)	Number of Events
Adverse events* n (%)	6	9	91	188	5	9	37	73	24	55	16	27	2	6	181 (67.5)	367
Adverse event related with biologic agent**	3	3	65	115	2	3	23	27	15	37	7	9	1	2	116 (43.3)	186
*Administration site reactions¨*	1	1	46	49	1	1	18	20	5	12	2	3	0	0	73 (27.2)	76
*Respiratory infections and other types of infection* *^+^*	1	1	22	30	1	1	1	1	5	5	0	0	0	0	30 (11.2)	38
*Nervous System* *^&^*	0	0	9	9	0	0	1	1	8	8	3	3	1	1	22(8.2)	22
*Cardiovascular*	0	0	3	11	0	0	2	4	1	5	1	1	1	1	8 (3.0)	22
*Metabolic*	0	0	3	5	0	0	1	1	1	1	0	0	0	0	5 (1.9)	7
*Gastrointestinal tract*	0	0	5	6	1	1	0	0	1	3	1	1	0	0	8 (3.0)	11
*Immune*	1	1	5	5	0	0	0	0	3	3	1	1	0	0	10 (3.7)	10
**SAE (n = 29)**																
*Inpatient* *Hospitalization*	0		10		1		3		1		0		0		15 (5.6)	
*Prolongation of existing hospitalization*	0		5		0		1		0		0		0		6 (2.2)	
*Life-threatening* *^&&^*	0		5		0		1		1		1		0		8 (3.0)	
**Withdraw due ADRs**	0		7		0		1		7		0		0		15 (5.6)	

*A patient could have more than one event for system.*

ADR, adverse drug reactions; SAE, serious adverse event.

^*^Adverse events ranked as possible, probable and definite.

^**^Adverse event ranked as only definite or probable.

¨Hypersensitivity reactions and cytokine-release syndrome.

^+^Opportunistic infections, infections of the urinary tract, infections of the skin, other systemic fungal infections and infection of the meninges.

^&^Headache and neuropathies.

^&&^Life-threatening: requires inpatient hospitalization or prolongation of existing hospitalization; creates persistent or significant disability/incapacity, or a congenital anomaly/birth defect’ (ICH, 1994).

**Table 3** shows the characteristics of population of the study with respect to predisposing factors of ADR and the association with ADRs. Overall, rituximab showed less risk to ADR [PR 0.42, 95% CI (0.18–0.96); *p* = 0.04] than other agents. The factors such as age, private healthcare assistance, provision of information about risk of ADRs, showed no association with ADRs.

**Table 3:** Association between predisposing factors and ADRs.

Characteristics	N-ADR	ADR*	PR 95% IC Unadjusted	P value	PR 95% IC Adjusted	P value
**Patients n (%)**	153	115				
**Age**						
19–59	81	70	1.00	–	1.00	–
60 or more	72	45	0.81 (0.56–1.19)	0.277	0.82 (0.55–1.21)	0.323
**Diagnostic (%)**						
Rheumatoid arthritis only	105	91	1.00	–	1.00	–
Psoriatic arthritis only	41	22	0.52 (0.23–1.19)	0.123	0.66 (0.28–1.55)	0.342
RA+PsA	7	2	0.89 (0.55–1.41)	0.609	1.07 (0.64–1.78)	0.790
**Comorbidity**						
None	72	58	1.00	–	1.00	–
1 or more	81	57	0.99 (0.69–1.42)	0.946	0.94 (0.64–1.38)	0.735
**Patient was guided about risk of medication**						
No	135	95	1.00	–	1.00	–
Yes	18	20	0.79 (0.49–1.27)	0.325	0.81 (0.49–1.32)	0.393
**Health insurance**						
Private	31	25	1.00	–	1.00	–
Public	122	90	0.95 (0.61–1.48)	0.824	0.91 (0.58–1.43)	0.694
**Biologic agent**						
adalimumab	63	64	1.00	–	1.00	–
abatacept	7	3	0.60 (0.19–1.90)	0.380	0.52 (0.16–1.68)	0.277
efalizumab	7	2	0.44 (0.11–1.80)	0.254	0.57 (0.13–2.46)	0.454
etanercept	29	23	0.88 (0.55–1.41)	0.592	0.86 (0.53–1.40)	0.531
infliximab	27	15	0.71 (0.40–1.24)	0.230	0.73 (0.40–1.31)	0.286
rituximab	18	7	0.56 (0.26–1.21)	0.140	0.42 (0.18–0.96)	0.044
tocilizumab	2	1	0.66 (0.09–4.77)	0.682	0.56 (0.08–4.05)	0.565
Concomitant use of drugs with biologic agents**						
No	50	31	1.00	–	1.00	–
Yes	103	84	1.17 (0.78–1.77)	0.446	1.15 (0.75–1.76)	0.534
**Concomitant use of DMARDs**						
No	74	50	1.00	–	1.00	–
Yes	79	65	1.02 (0.61–1.70)	0.935	0.98 (0.58–1.67)	0.944
**Duration use of biologic agents (months)**						
6 to 12 months	24	16	1.00	–	1.00	–
13 months or more	129	99	1.08 (0.64–1.83)	0.777	0.95 (0.55–1.62)	0.839

*ADR ranked as definite or probable.

Adjusted to: age, comorbidity and concomitant use of others drugs.

**DMARDs not included.

**Table 4** presents the clinical follow-up and outcome in patients with on-going biologic treatments. One hundred fifty-one (95.6%) patients visited a doctor at least once a year, however, 48 patient (30.4%) did not undergo the laboratory tests (complete blood count, liver function test, reactive protein test), while 75 (47.5%) did get radiography done, whereas 58 (36.7%) patients had at least two medical consultations, underwent a laboratory blood test at least once, and had a radiography examination once a year.

**Table 4:** Clinical follow up and outcome judgment in patients with psoriatic arthritis and rheumatoid arthritis still taking biologics.

Outcomes	abatacept (n = 6) n (%)	adalimumab (n = 75) n (%)	etanercept (n = 39) n (%)	infliximab (n = 16) n (%)	rituximab (n = 19) n (%)	tocilizumab (n = 3) n (%)
**Annual Review**						
**A) Consults¹**	6 (100.0)	69 (92.0)	38 (97.4)	16 (100.0)	19 (100.0)	3 (100.0)
**B) Lab exams²**	3 (50.0)	54 (72.0)	25 (64.1)	11 (68.7)	14 (73.7)	3 (100.0)
CBC	4 (66.7)	71 (94.7)	36 (92.3)	14 (87.5)	16 (84.2)	3 (100.0)
Liver function test	4 (66.7)	59 (78.7)	30 (76.9)	12 (75.0)	16 (84.2)	3 (100.0)
CRP test	3 (50.0)	58 (77.3)	26 (66.7)	11 (68.7)	14 (73.7)	3 (100.0)
**C) Radiograph³**	3 (50.0)	43 (57.3)	19 (48.7)	7 (43.7)	10 (52.6)	1 (33.3)
**Adequate clinical monitoring**						
D) A + B + C	3 (50.0)	33 (44.0)	9 (23.1)	5 (31.2)	7 (36.8)	1 (33.3)

*efalizumab was removed from the market in 2009.*

¹At least two annual medical consults.

²Laboratory blood tests performed at least once a year (CBC – Complete Blood Count; Liver function tests; CRP test – C-Reactive Protein test).

³Adiography performed at least once a year.

**Clinical follow up was checked in pts still taking the medication during the interview (n = 158).

## Discussion

### Main Findings

The typical long-term users of biological drugs in this study were women aged 19–59 years, with one or more comorbidities, using biological drugs for 13–36 months and having access to public healthcare. Around 40% of the patients using biologics drugs for a long-term, had one or more ADR related to these agents, with 1.6 events per patient. The most common ADRs were administration site reactions, infections and symptoms related to nervous system (headache and neuropathic pain). The occurrence of SAEs was less than 10% of all patients. Nevertheless, the majority of SAEs did not lead to drug discontinuation. Notably, no case of tuberculosis or mortality were detected. None of other risk factors studied were associated with ADRs. Adalimumab was the biological agent most used for the majority of patients and ADRs related with this drug included serious infections disease and injections site reactions. The use of rituximab was related with lower risk of ADR than other agents. Complete clinical follow-up was done by 36.7% of patients, implying that remain the patients, despite receiving government medicines failed to follow-up, according to the official Brazilian guidelines.

### Relation to Prior Literature

Biologicals have become potent and effective therapeutic alternative for many inflammatory and autoimmune diseases like RA and PsA, focus of this cohort population. Their direct and focused effect makes them superior to classic immunosuppressive, whose use is frequently limited by undesirable and often severe generalized adverse effect. Biologic agents targeting specific immune mediators have emerged as other treatment option for patients with RA, PsA and others immune disease who are unresponsive to, or intolerant of, non-biologic systemic agents. (Yazici, 2018; Michet, 2018).

Furthermore, conventional treatments for PsA have limited efficacy for nail disease, enthesitis or axial involvement, and some are unable to control moderate and severe peripheral joint and skin disease (Yazici, 2018). The introduction of biologic treatments offered the possibility of controlling multiple aspects of these diseases using a single drug, minimizing the need for additional therapies (Elyoussfi et al., 2016).

Although several biologics have demonstrated good efficacy and tolerability in short-term trials, treatment guidelines recommend them as third line therapies due to a relative lack of long-term safety data. Here, we have reviewed the long-term (>6 months) safety data. In our study, 35% of patients used biological agents for more than 37 months, reflecting the real scenario of its long-term use in Brazil.

Evidently, the harms of biologics must be balanced against their use benefits, when making a risk–benefit assessment of its use for a patient with systemic autoimmune conditions such as RA or PsA. Patients and physicians worry about risks including not only common ADRs such injection site reactions but also infections that may be less common.

The clinical representation of ADR to biologicals can be ambiguous with regard to pathogenesis of the reaction, because different pathomechanisms may lead to similar symptoms. This is especially important for infusion reactions, whereby no clear clinical distinction between allergic, IgE-mediated, and the more frequent no allergic, most probably complement-mediated reactions are possible. In our study, despite the long-term use of biological agents, mainly the anti-TNFα drugs (infliximab, etanercept and adalimumab) they still have reactions related with administrations. Infusions/Injections site reactions are a major complication of all anti-TNFα drugs with studies showing an incidence rate 3–40% (Henderson Berg and Carrasco, 2017).

The most common cutaneous side effects are injection site reactions, which are often defined as a constellation of symptoms, including swelling, erythema, pruritus, and pain around the site of injection (Zeltser et al., 2001; Clarke, 2010; Scherer et al., 2010). Administration site reactions can be divided into two types according to their mechanism of action: i. Type α - irritative reactions (immediate) commonly at the injection sites of subcutaneously administered biologics caused by proinflammatory actions of the substances (Corominas et al., 2014); ii. the Type hypersensitivity reactions categorized into Types I–IV, which are induced by IgE, IgG/IgM, complement or T-cells. Injection site reaction after etanercept injection produce a T-cell-mediated delayed-type hypersensitivity reaction, as approximately 8% of patients developed “recall injection site reaction,” reactions at sites were medication was previously injected (Scherer et al., 2010).

The injections site reactions with etanercept, can occur in up to 37% of patients and characteristically these reactions consist of mild to moderate erythema, pain, pruritus and edema immediately evident or appear within 24–48 h and the mean duration of the reaction is 3–5 d and there is a gradual decrease in frequency and severity with continuation of injections (Kim et al., 2015; Murdaca et al., 2012). This didn’t happen in the population of this cohort. Patients using adalimumab, etanercept and infliximab more than 6 months in our cohort had 36, 34 and 11% of infusion/injections site reactions. Data from the Hong Kong Biologics Registry that followed up 1,345 patients from 2005–2013 that reported the most frequent SAE (per 100-paties-year) was infusion/injection site reaction (0.75) (Mok et al., 2014).

We found that inappropriate injection techniques, injection close to blood vessels, the chemical and physical properties of the injected drug and a reaction to the vehicle component are several causes described in the literature resulting in irritative reactions (Corominas et al., 2014).

Severe infusion reactions, such as angioedema and shock, have been reported in patients under infliximab therapy. As infliximab is a chimeric human/mouse anti- TNF-α antibody, it may induce the synthesis of neutralizing antibodies which could reduce the efficacy of the drug. Therefore, methotrexate is usually co-administered to control both the rheumatic disease and the development of neutralizing antibodies (Murdaca et al., 2013). In spite of its fully human sequence, the production of antibodies to adalimumab has been also reported, which may reduce the efficacy of the drug and induce the development of adverse drug reactions and exanthema (Murdaca et al., 2012).

However, in the majority of cases the injections site reaction with adalimumab in our study were mild-to-moderate severity, and do not necessitated drug discontinuation.

Studies comparing the intravenous and subcutaneous route of administration of these two agents did not show any difference in clinical efficacy and safety, except that injection site reactions were more common with the subcutaneous access which is the case of adalimumab and etanercept use differently of infliximab that is indicate to infusion use (Gabay et al., 2013).

Few biologics are associated with a higher rate of some of the ADRs than others, and potential SAEs with short-term and long-term use (Singh et al., 2011). The safety of rituximab was consistent with earlier findings, which indicated that there was no increase in ADRs or SAE with its prolonged use (>5 years) (Van Vollenhoven et al., 2013; Winthrop et al., 2018).

This cohort is related to the use of long-term biologicals, which shows that the adverse effects may be different from those studies that indicate short-term adverse effects. The biologic use can increase the risk of serious infections in the first months of treatment with respect to disease-modifying antirheumatic drugs (DMARDs). Data from the British Society for Rheumatology Biologics Register (BSRBR) comparing the risk of serious infections between TNFi-treated patients and traditional DMARD-treated patients showed that the risk of serious infections with TNFi was increased in the first 6 months of initiating therapy for RA and that this risk was higher compared to traditional DMARDs (Galloway et al., 2011).

Another largest observational studies of infection in patients with autoimmune diseases found that compared to traditional DMARDs, the initiation of TNF-alpha antagonists was not associated with an increased risk of hospitalizations for serious infections (Grijalva et al., 2011).

Serious infections (such as infections requiring hospitalization or intravenous administration of antibiotics, opportunistic infections, including tuberculosis, systemic fungal infections and herpes zoster) were uncommon in population currently using the biological agents for more than six months.

Moreover, long-term use of biological could be related with non-serious infections, mainly of the upper respiratory tract. This ADR were common among the users of biologics in our study, endorsing the findings from earlier studies (Salliot et al., 2007; Winthrop et al., 2018).

Emerging data also suggest that the incidence of serious infections is dependent on, past history of serious infections, corticosteroids anti-inflammatory doses, and older age as important predictors of risk of serious infections in patients treated with biologics. The duration of treatment is also an important risk factor with the highest rate being observed during the first months of therapy and the risk of infection decreases over time (Singh, 2016). Our study included patients who used biologics for a prolonged period, which could explain the reduced number of opportunistic infections. No case of tuberculosis was reported.

A study in Latin America showed that the risk of serious infections may vary depending on the region and the characteristics of the patient (Ranza et al., 2019). This study compared a database not available publicly (BIOBADABRASIL) from Brazil versus a database of Argentina (BIOBADASAR) and showed that the risk of infections in Brazil was decreasing over time, corroborating our findings.

Nowadays, safety profile has changed mainly because we know more about the disease and about biologic agent. Furthermore, not only the function of these composites has to be understood, but also the subjacent immunology (which is very complex). Therefore, current patients do have less prolonged and severe chronic inflammation, key element for decrease of cardiovascular events or malignancy risk.

The delayed reactions related to dysfunction of the cellular response such as autoimmunity or cancer, may appear after many months or years of the cessation of the biologic therapy (Mazurek and Jahnz-Rozyk, 2012). However, recent cohort study showed no evidence of change in risk of solid cancer with increasing exposure to biologics in the first five years (Mercer et al., 2015).

The characteristics of the population of this study are similar to those found in studies carried out in Europe and USA (Sfriso et al., 2009; Schneeweiss et al., 2017). The most of patients were women, age around 50–55 years old, with at least one comorbidity.

Comorbidities in our study also were similar to those observed in other studies (Ruiz et al., 2014; Deus et al., 2015; Camargo et al., 2016; Ryan et al., 2011). Patients with RA had more cardiovascular diseases, metabolic diseases like type 2 diabetes mellitus and obesity, and muscle pain rendering in work disabilities. Patients with PsA also had more cardiovascular disease, metabolic disease, inflammatory bowel disease and autoimmune ophthalmic disease. Comorbidities in this study were not related to increase in risk of ADR, as found in patients with only psoriasis in other study (Lopes et al., 2014).

Considering the high costs of biologic therapies and their adverse events profile in long-term use, the follow up of the patients and individual monitoring is essential (Mazurek and Jahnz-Rozyk, 2012). The big problem here with the use of biologics is that the prescription is made by a private doctor and accessibility to the medicine is dependent on public service, disallowing appropriate monitoring.

Furthermore, the wide use of biological agents in modern medicine is a challenge in clinical practices, as it is a case of how fast new therapeutic principles based on novel knowledge and modern techniques can enter clinical practice, and that constant learning is required. Their use often requires a special knowledge and familiarity with the disease to be treated. Scientific data shows that ADRs to these drugs are clinically very heterogeneous. It makes clear, the monitoring of them seems essential (Pichler, 2006; Zemkova et al., 2007). Patients on biologic therapy should be monitored closely with routine blood tests, regular doctor’s visit and outcome measures about effectiveness (considering the response to therapy), safety (presence of ADRs and SAEs) and quality of health for prolonged period (Emer et al., 2010; RCN, 2017).

There is a lack of data regarding the best interval for monitoring the patients. We would like to emphasize that we have adopted the recommendations of the Brazilian Clinical Protocols of the time. These are official government protocols, since these medications are only provided in government pharmacies. Most of the patients had medical consultations, and laboratory tests for liver function and blood cell counts, at least annually, according to the recommendations of several guidelines from England, Brazil, Canada, etc. (BRAZIL, 2006; Smith et al., 2009; Bykerk et al., 2012; BRAZIL, 2014; Michet, 2018, Coates et al., 2016; Gossec et al., 2016). The recommendations for radiography vary according to the adopted guideline. Usually, radiographic assessments are encouraged according to clinical manifestations and discretion of physician (Ritchlin et al., 2008). In this study, we decided to follow the Brazilian guideline, and the radiography was assumed as a test for monitoring the progression of tuberculosis.

The most appropriate way to monitor disease activity in PsA is under defined (Gossec et al., 2016). The recommended core set for PsA comprises of peripheral joints, pain, physical and global function assessment, quality of life and fatigue (Kimball et al., 2008). It is also recommended that radiographic monitoring for erosions and osteolysis of the hands and feet be done annually (Michet, 2018).

The effectiveness of the biologics in RA and PsA is unquestionable, but their association with potentially adverse effects can doubt the benefit risk ratio. Therefore, closer monitoring and education of patient and their caregivers about the nature of their condition, benefits and risks of treatment are essential for improving treatment outcome and overall patient satisfaction (Emer et al., 2010). The patients need to be advised to report any worsening of symptoms (neurological, cardiac, pulmonary, skin, uveitis and/or malignancies), avoid exposure to potential risk factors for infection, and to promptly communicate to their physician possible signs and symptoms of infection (RCN, 2017).

### Strengths and Limitations

The strengths of this study included an extensive questionnaire, with questions about previous treatments, diagnosis, comorbidities, and if any adverse effects and its interference with the current treatment. Besides that, our sample size was relatively large, we contacted 305 patients and interviewed 268. Our data was checked twice, as we obtained data about use of medication from the pharmacy records and then confirmed the information from patient interview to ensure its accuracy.

This registry study has mounting importance in medical research and decision-making processes. In fact, despite the inherent limitations of such studies, including the lack of randomization, the relatively high frequency of missing data, and the presence of patients with different diseases, registries usually include larger populations than clinical trials, and therefore have a higher power to detect rare adverse events. In addition, registry studies may better reflect clinical practice with respect to randomized clinical trials, whose results may not immediately be extended to “real-life”. Today, several registries in Europe are collecting data on the use of biological Drugs: ARTIS (Antirheumatic Therapies In Sweden), BIOBADASER (Base de Datos de Productos Biológicos de la Sociedad Española de Reumatología, Spain), BSRBR (British Society for Rheumatology Biologics Register, United Kingdon), DANBIO (Danish Database for Biological Therapies in Rheumatology, Denmark), RATIO (Research Axed on Tolerance of Biotherapies, France) etc. Though most patients included in these registries are taking biologic agents for the treatment of RA/PsA, the safety information gained from these sources can be applied in clinical practices (Nikiphorou et al., 2017).

Brazil have had limited initiatives to build up an important national database linking with important clinical outcomes in RA/PsA/Psoriasis. There are databases restricted to a private insurance sustained by societies of rheumatologist and funding by pharmaceutical industry. An example of this is a multicentre prospective observational cohort in Brazil is the REAL (Rheumatoid arthritis in real life) that is following up 1,300 patients from 11 canters in 4 regions from Brazil, since 2015, funding by Bristol-Myers Squibb, Eli Lily and others pharmaceutical industries (Da Rocha Castelar-Pinheiro et al., 2018). Our study is one of the few studies in Brazil that had access to patients in public health sector, without any conflict of interest with long term follow-up.

Recall bias may have led to obtaining inaccurate information about previous treatments, side effects, and clinical monitoring. We try to reduce this limitation by only asking about the clinical monitoring to those who were still using the biological agents. Another limitation is the fact that ADR were not validated by a treating physician. However, some studies point that data reported by patients are potentially important to support and improve the care (Dawson et al., 2010; Nelson et al., 2015). Furthermore, in many countries, pharmacists are recognized as one of the most important healthcare providers in ADR reporting like in the Netherlands, Spain, Portugal, and Korea (Van Grootheest et al., 2004; Kim et al., 2010; Yu et al., 2016).

### Implications

This study showed the association of biological agents with ADRs, withdrawals due to adverse events and SAEs in long-term use. Patients using biological agents must be aware of these risks and should be subjected to careful monitoring throughout the treatment to prevent or at least treat a possible ADR. Our data suggests that, patients need comorbidity warning of possible adverse events and recommended enhanced surveillance.

In the long-term, the possible risk of SAE requires caution and further monitoring and investigation. Therefore, review and further investigations of their safety are warranted.

## Ethics Statement

The protocol was authorized by the Health State Department and approved by the ethics committee for clinical research of University of Sorocaba on August 17, 2009, with protocol number 011/2009.

## Author Contributions

LCL had the original idea and reviewed all of steps of the manuscript conception. MCC, IAC and MSNS collected the data. SBF, BCAB and FSDF performed the adverse reactions analysis and cross-checked the data. MTS performed data statistical analysis. BCAB and IF drafted the manuscript.

## Funding

The authors have received support from FAPESP (Foundation for Research Support of the State of São Paulo, process number 2009/530841).

## Conflict of Interest Statement

The authors declare that the research was conducted in the absence of any commercial or financial relationships that could be construed as a potential conflict of interest.

## References

[B1] BRAZIL (2006). Portaria n°. 66, de 6 de novembro de 2006. Health Tecnology and Strategy Drugs Secretariat. Clinical Protocol and Guidelines- Rheumatoid Arthrist Diário Oficial da União. Available at: http://bvsms.saude.gov.br/bvs/saudelegis/sctie/2006/prt0066_01_11_2006_comp.html (Acessed January 06 2019).

[B2] BRAZIL (2014). Artrite Psoriática. Protocolo Clínico e Diretrizes Terapêuticas. Portaria SAS/MS n° 1.204, de 4 de novembro de 2014. Available at: http://conitec.gov.br/images/Protocolos/Artrite-Psoriaca.pdf (Accessed January 19, 2019).

[B3] Bykerk V. P. Akhavan P. Hazlewood G. S. Schieir O. Dooley A. Haraoui B. (2012). Canadian Rheumatology Association recommendations for pharmacological management of rheumatoid arthritis with traditional and biologic disease-modifying antirheumatic drugs. J. Rheumatol. 39 (8), 1559–1582. 10.3899/jrheum.110207 21921096

[B4] Camargo I. A. Barros B. C. A. Nascimento Silveira M. S. Osorio-de-Castro C. G. S. Guyatt G. Lopes L. C. (2016). Gap between official guidelines and clinical practice for the treatment of rheumatoid arthritis in São Paulo, Brazil. Clin. Ther. 38, 1122–1133. 10.1016/j.clinthera.2016.02.020 26976223

[B5] Chen Y. F. Jobanputra P. Barton P. Jowett S. Bryan S. Clark W. (2006). A systematic review of the effectiveness of adalimumab, etanercept and infliximab for the treatment of rheumatoid arthritis in adults and an economic evaluation of their cost-effectiveness. Health Technol. Assess. 10 (42), iii–iv, xi–xiii, 1–229. 10.3310/hta10420 17049139

[B6] Clarke J. B. (2010). Mechanisms of adverse drug reactions to biologics. Handb. Exp. Pharmacol. 196, 453–474. 10.1007/978-3-642-00663-0_16 20020272

[B7] Coates L. C. Tillett W. Chandler D. Helliwell P. S. Korendowych E. Kyle S. (2013). The 2012 BSR and BHPR guideline for the treatment of psoriatic arthritis with biologics. Rheumatol. (Oxf. Engl.) 52 (10), 1754–1757. 10.1093/rheumatology/ket187 23887065

[B8] Coates L. C. Kavanaugh A. Mease P. J. Soriano E. R. Laura Acosta-Felquer M. Armstrong A. W. (2016). Group for Research and Assessment of Psoriasis and Psoriatic Arthritis 2015 Treatment Recommendations for Psoriatic Arthritis. Arthritis Rheumatol. 68 (5), 1060–1071. 10.1002/art.39573 26749174

[B9] Corominas M. Gastaminza G. Lobera T. (2014). Hypersensitivity reactions to biological drugs. J. Invest. Allergol. Clin. Immunol. 24 (4), 212–225. 25219103

[B10] Curtis J. R. Xie F. Chen L. Baddley J. W. Beukelman T. Saag K. G. (2011). The comparative risk of serious infections among rheumatoid arthritis patients starting or switching biological agents. Ann. Rheum. Dis. 70 (8), 1401–1406. 10.1136/ard.2010.146365 21586439 PMC3128235

[B11] Da Rocha Castelar-Pinheiro G. Vargas-Santos A. B. de Albuquerque C. P. Bertolo M. B. Junior P. L. Giorgi R. D. N. (2018). The REAL study: a nationwide prospective study of rheumatoid arthritis in Brazil. Adv. Rheumatol. (Lond. Engl.) 58 (1), 9. 10.1186/s42358-018-0017-9 30657089

[B12] Dawson J. Doll H. Fitzpatrick R. Jenkinson C. Carr A. J. (2010). The routine use of patient reported outcome measures in healthcare settings. BMJ 340, c186. 10.1136/bmj.c186 20083546

[B13] Deighton C. O’Mahony R. Tosh J. Turner C. Rudolf M. (2009). Management of rheumatoid arthritis: summary of NICE guidance. BMJ Br. Med. J. 338, b702. 10.1136/bmj.b702 19289413 PMC3266846

[B14] Deus R. S. Ferraz A. L. Oesterreich S. A. Schmitz W. O. Shinzato M. M. (2015). Caracterização de pacientes com artrite reumatoide quanto a fatores de risco para doenças vasculares cardíacas no Mato Grosso do Sul. Rev. Bras. Reumatol. 55 (6), 493–500. 10.1016/j.rbr.2015.02.001 26362702

[B15] Elyoussfi S. Thomas B. J. Ciurtin C. (2016). Tailored treatment options for patients with psoriatic arthritis and psoriasis: review of established and new biologic and small molecule therapies. Rheumatol. Int. 36 (5), 603–612. 10.1007/s00296-016-3436-0 26892034 PMC4839046

[B16] Emer J. J. Frankel A. Zeichner J. A. (2010). A practical approach to monitoring patients on biological agents for the treatment of psoriasis. J. Clin. Aesthet. Dermatol. 3 (8), 20–26. PMC2945861 20877538

[B17] Fowler F. J. Jr. Epstein A. Weingart S. N. Annas C. L. Bolcic-Jankovic D. Clarridge B. (2008). Adverse events during hospitalization: results of a patient survey. Jt. Comm. J. Qual. Patient Saf. Jt. Comm. Resour. 34 (10), 583–590. 10.1016/S1553-7250(08)34073-2 PMC13410503 18947118

[B18] Gabay C. Emery P. van Vollenhoven R. Dikranian A. Alten R. Pavelka K. (2013). Tocilizumab monotherapy versus adalimumab monotherapy for treatment of rheumatoid arthritis (ADACTA): a randomised, double-blind, controlled phase 4 trial. Lancet (Lond. Engl.) 381 (9877), 1541–1550. 10.1016/S0140-6736(13)60250-0 23515142

[B19] Galloway J. B. Hyrich K. L. Mercer L. K. Dixon W. G. Fu B. Ustianowski A. P. (2011). Anti-TNF therapy is associated with an increased risk of serious infections in patients with rheumatoid arthritis especially in the first 6 months of treatment: updated results from the British Society for Rheumatology Biologics Register with special emphasis on risks in the elderly. Rheumatol. (Oxf. Engl.) 50 (1), 124–131. 10.1093/rheumatology/keq242 PMC3105607 20675706

[B20] Girolomoni G. Altomare G. Ayala F. Berardesca E. Calzavara-Pinton P. Chimenti S. (2012). Safety of anti-TNFα agents in the treatment of psoriasis and psoriatic arthritis. Immunopharmacol. Immunotoxicol. 34 (4), 548–560. 10.3109/08923973.2011.653646 22296031

[B21] Gonzalez-Alvaro I. Martinez-Fernandez C. Dorantes-Calderon B. Garcia-Vicuna R. Hernandez-Cruz B. Herrero-Ambrosio A. (2015). Spanish Rheumatology Society and Hospital Pharmacy Society Consensus on recommendations for biologics optimization in patients with rheumatoid arthritis, ankylosing spondylitis and psoriatic arthritis. Rheumatol. (Oxf.) 54 (7), 1200–1209. 10.1093/rheumatology/keu461 PMC4473767 25526976

[B22] Gossec L. Smolen J. S. Ramiro S. de Wit M. Cutolo M. Dougados M. (2016). European League Against Rheumatism (EULAR) recommendations for the management of psoriatic arthritis with pharmacological therapies: 2015 update. Ann. Rheum. Dis. 75 (3), 499–510. 10.1136/annrheumdis-2015-208337 26644232

[B23] Grijalva C. G. Chen L. Delzell E. Baddley J. W. Beukelman T. Winthrop K. L. (2011). Initiation of tumor necrosis factor-alpha antagonists and the risk of hospitalization for infection in patients with autoimmune diseases. Jama 306 (21), 2331–2339. 10.1001/jama.2011.1692 22056398 PMC3428224

[B24] Hausmann O. V. Seitz M. Villiger P. M. Pichler W. J. (2010). The complex clinical picture of side effects to biologicals. Med. Clin. North America 94 (4), 791–804, xi–ii. 10.1016/j.mcna.2010.03.001 20609863

[B25] Henderson Berg M. H. Carrasco D. (2017). Injection Site Reactions to Biologic Agents Used in Psoriasis and Psoriatic Arthritis. J. Drugs Dermatol. JDD 16 (7), 695–698. 28697222

[B26] Hibbard J. H. Peters E. Slovic P. Tusler M. (2005). Can patients be part of the solution? Views on their role in preventing medical errors. Med. Care Res. Rev. MCRR 62 (5), 601–616. 10.1177/1077558705279313 16177460

[B27] International Council on Harmonisation (1994). E2A Clinical safety data management: definition and standards for expedited reporting. 12 p. Available at: https://www.ich.org/fileadmin/Public_Web_Site/ICH_Products/Guidelines/Efficacy/E2A/Step4/E2A_Guideline.pdf (Accessed June 14 2019).

[B28] Karmacharya P. Poudel D. R. Pathak R. Donato A. A. Ghimire S. Giri S. (2015). Rituximab-induced serum sickness: a systematic review. Semin. Arthritis Rheum. 45 (3), 334–340. 10.1016/j.semarthrit.2015.06.014 26199061

[B29] Katz U. Zandman-Goddard G. (2010). Drug-induced lupus: an update. Autoimmun. Rev. 10 (1), 46–50. 10.1016/j.autrev.2010.07.005 20656071

[B30] Kim W. B. Marinas J. E. Qiang J. Shahbaz A. Greaves S. Yeung J. (2015). Adverse events resulting in withdrawal of biologic therapy for psoriasis in real-world clinical practice: a Canadian multicenter retrospective study. J. Am. Acad. Dermatol. 73 (2), 237–241. 10.1016/j.jaad.2015.04.023 26026334

[B31] Kim J. Y. Ha J.-H. Kim B.-R. Jang J. Hwang M. Park H.-J. (2010). Analysis of Characteristics about Spontaneous Reporting—Reported in 2008. JPERM 3, 23–31.

[B32] Kimball A. B. Gladman D. Gelfand J. M. Gordon K. Horn E. J. Korman N. J. (2008). National Psoriasis Foundation clinical consensus on psoriasis comorbidities and recommendations for screening. J. Am. Acad. Dermatol. 58 (6), 1031–1042. 10.1016/j.jaad.2008.01.006 18313171 PMC3716382

[B33] Kuzel A. J. Woolf S. H. Gilchrist V. J. Engel J. D. LaVeist T. A. Vincent C. (2004). Patient reports of preventable problems and harms in primary health care. Ann. Family Med. 2 (4), 333–340. 10.1370/afm.220 PMC1466690 15335132

[B34] Lee S. J. Kavanaugh A. (2005). Adverse reactions to biologic agents: focus on autoimmune disease therapies. J. Allergy Clin. Immunol. 116 (4), 900–905. 10.1016/j.jaci.2005.03.028 16210067

[B35] Lopes L. C. Silveira M. S. de Camargo M. C. de Camargo I. A. Luz T. C. Osorio-de-Castro C. G. (2014). Patient reports of the frequency and severity of adverse reactions associated with biological agents prescribed for psoriasis in Brazil. Exp. Opin. Drug Safety 13 (9), 1155–1163. 10.1517/14740338.2014.942219 25078511

[B36] Mariette X. Matucci-Cerinic M. Pavelka K. Taylor P. van Vollenhoven R. Heatley R. (2011). Malignancies associated with tumour necrosis factor inhibitors in registries and prospective observational studies: a systematic review and meta-analysis. Ann. Rheum. Dis. 70 (11), 1895–1904. 10.1136/ard.2010.149419 21885875

[B37] Mazurek J. Jahnz-Rozyk K. (2012). The variety of types of adverse side-effects during treatment with biological drugs. Int. Rev. Allergol. Clin. Immunol. Family Med. 18, 34–40.

[B38] Mercer L. K. Lunt M. Low A. L. Dixon W. G. Watson K. D. Symmons D. P. (2015). Risk of solid cancer in patients exposed to anti-tumour necrosis factor therapy: results from the British Society for Rheumatology Biologics Register for Rheumatoid Arthritis. Ann. Rheum. Dis. 74 (6), 1087–1093. 10.1136/annrheumdis-2013-204851 24685910 PMC4431340

[B39] Michet C.J. (last update Mar 2018). Psoriatic arthritis. BMJ Best Practice. Available at: bestpractice.bmj.com (Accessed 04/10/2019).

[B40] Mok C. C. Chan K. Y. Lee K. L. Tam L. S. Lee K. W. (2014). Factors associated with withdrawal of the anti-TNFalpha biologics in the treatment of rheumatic diseases: data from the Hong Kong Biologics Registry. Int. J. Rheum. Dis. 17 Suppl: 3, 1–8. 10.1111/1756-185X.12264 24382315

[B41] Murdaca G. Colombo B. M. Cagnati P. Gulli R. Spano F. Puppo F. (2012). Update upon efficacy and safety of TNF-alpha inhibitors. Exp. Opin. Drug Safety 11 (1), 1–5. 10.1517/14740338.2012.630388 22010813

[B42] Murdaca G. Spano F. Puppo F. (2013). Selective TNF-alpha inhibitor-induced injection site reactions. Exp. Opin. Drug Safety 12 (2), 187–193. 10.1517/14740338.2013.755957 23330811

[B43] Murdaca G. Spano F. Contatore M. Guastalla A. Penza E. Magnani O. (2015). Infection risk associated with anti-TNF-alpha agents: a review. Exp. Opin. Drug Safety 14 (4), 571–582. 10.1517/14740338.2015.1009036 25630559

[B44] Naranjo C. A. Busto U. Sellers E. M. Sandor P. Ruiz I. Roberts E. A. (1981). A method for estimating the probability of adverse drug reactions. Clin. Pharmacol. Ther. 30 (2), 239–245. 10.1038/clpt.1981.154 7249508

[B45] Nelson E. C. Eftimovska E. Lind C. Hager A. Wasson J. H. Lindblad S. (2015). Patient reported outcome measures in practice. BMJ 350, g7818. 10.1136/bmj.g7818 25670183

[B46] NICE (2012). National Clinical Guideline Centre (UK). National Institute for Health and Clinical Excellence: Guidance. Psoriasis: Assessment and Management of Psoriasis. London: Royal College of Physicians (UK) 2012. Available at: https://www.ncbi.nlm.nih.gov/books/NBK247829/ (Accessed 01/06/2019).

[B47] Nikiphorou E. Buch M. H. Hyrich K. L. (2017). Biologics registers in RA: methodological aspects, current role and future applications. Nat. Rev. Rheumatol. 13 (8), 503–510. 10.1038/nrrheum.2017.81 28569267

[B48] Pichler W. J. (2006). Adverse side-effects to biological agents. Allergy 61 (8), 912–920. 10.1111/j.1398-9995.2006.01058.x 16867042

[B49] Ranza R. de la Vega M. C. Laurindo I. M. M. Gómez M. G. Titton D. C. Kakehasi A. M. (2019). Changing rate of serious infections in biologic-exposed rheumatoid arthritis patients. Clin. Rheumatol. 38 (8), 2129–2139. 10.1007/s10067-019-04516-2 31016578

[B50] RCN (2017). Royal College of Nursing. Assessing, Managing and Monitoring Biologic Therapies for Inflammatory Arthritis. RCN Guidance for Rheumatology Practitioners Fourth edition 96 p. Available at: https://www.rcn.org.uk/-/media/royal-college-of nursing/documents/publications/2017/september/pdf-005579.pdf (Accessed 01/19/2019).

[B51] Ritchlin C. T. Kavanaugh A. Gladman D. D. Mease P. J. Helliwell P. Boehncke W. H. (2008). Treatment recommendations for psoriatic arthritis. Ann. Rheum. Dis. 68 (9), 1387–1394. 10.1136/ard.2008.094946 18952643 PMC2719080

[B52] Rosman Z. Shoenfeld Y. Zandman-Goddard G. (2013). Biologic therapy for autoimmune diseases: an update. BMC Med. 11, 88. 10.1186/1741-7015-11-88 23557513 PMC3616818

[B53] Ruiz D. G. Azevedo M. N. L. Santos O. L. R. (2014). Caracterização clínica de pacientes com artrite psoriásica. Rev. Soc. Bras. Clin. Med. 12 (2), 1–3.

[B54] Ryan C. Leonardi C. L. Krueger J. G. Kimball A. B. Strober B. E. Gordon K. B. (2011). Association between biologic therapies for chronic plaque psoriasis and cardiovascular events: a meta-analysis of randomized controlled trials. Jama 306 (8), 864–871. 10.1001/jama.2011.1211 21862748

[B55] Salliot C. Gossec L. Ruyssen-Witrand A. Luc M. Duclos M. Guignard S. (2007). Infections during tumour necrosis factor — αblocker therapy for rheumatic diseases in daily practice: a systematic retrospective study of 709 patients. Rheumatol. (Oxf.) 46 (2), 327–334. 10.1093/rheumatology/kel236 16880188

[B56] Scherer K. Spoerl D. Bircher A. J. (2010). Adverse drug reactions to biologics. J. Dtsch. Dermatol. Ges. J. Ger. Soc. Dermatol. JDDG 8 (6), 411–426. 10.1111/j.1610-0387.2010.07339.x 20136676

[B57] Schneeweiss M. Merola J. F. Karlson E. W. Solomon D. H. (2017). Rationale and desig of the Brigham cohort for psoriasis and psoriatic arthritis registy (COPPAR). BMC Dermatology 17 (1), 11, 1–9. 10.1186/s12895-017-0063-8 28814312 PMC5559864

[B58] Sfriso P. Salaffi F. Montecucco C. M. Bombardieri S. Todesco S. (2009). MonitorNet: the Italian multi-centre observational study aimed at estimating the risk/benefit profile of biologic agents in real-world rheumatology practice. Reumatismo 61 (2), 132–139. 10.4081/reumatismo.2009.132 19633800

[B59] Silveira M. S. de Camargo I. A. Osorio-de-Castro C. G. Barberato-Filho S. Del Fiol F de S. Guyatt G. (2014). Adherence to guidelines in the use of biological agents to treat psoriasis in Brazil. BMJ Open 4 (3), e004179. 10.1136/bmjopen-2013-004179 PMC3948458 24598304

[B60] Smith C. H. Anstey A. V. Barker J. N. Burden A. D. Chalmers R. J. Chandler D. A. (2009). British Association of Dermatologists’ guidelines for biologic interventions for psoriasis 2009. Br. J. Dermatol. 161 (5), 987–1019. 10.1111/j.1365-2133.2009.09505.x 19857207

[B61] Singh J. A. Wells G. A. Christensen R. Tanjong Ghogomu E. Maxwell L. Macdonald J. K. (2011). Adverse effects of biologics: a network meta-analysis and Cochrane overview. Cochrane database Syst. Rev. 2, CD008794. 10.1002/14651858.CD008794.pub2 PMC7173749 21328309

[B62] Singh J. A. (2016). Infections With Biologics in Rheumatoid Arthritis and Related Conditions: a Scoping Review of Serious or Hospitalized Infections in Observational Studies. Curr. Rheumatol. Rep. 18 (10), 61. 10.1007/s11926-016-0609-5 27613285

[B63] Van Grootheest K. Olsson S. Couper M. de Jong-van den Berg L. (2004). Pharmacists’ role in reporting adverse drug reactions in an international perspective. Pharmacoepidemiol. Drug Saf. 13 (7), 457–464. 10.1002/pds.897 15269929

[B64] Van Vollenhoven R. F. Emery P. Bingham C. O. Keystone E. C. Fleischmann R. M. Furst D. E. (2013). Long-term safety of rituximab in rheumatoid arthritis: 9.5-year follow-up of the global clinical trial programme with a focus on adverse events of interest in RA patients. Ann. Rheum. Dis. 72 (9), 1496–1502. 10.1136/annrheumdis-2012-201956 23136242 PMC3756452

[B65] WHO (2002) World Health Organization. Department of Essential Drugs and Medicines Policy. Safety of medicines. A guide to detecting and reporting adverse drug reactions. Geneva: World Health Organization, 20 p. Available at: http://apps.who.int/iris/bitstream/handle/10665/67378/WHO_EDM_QSM_2002.2.pdf;jsessionid=F7D0B632670A3407829D860782550B58?sequence=1 (Accessed 01/06/2019).

[B66] Winthrop K. L. Saag K. Cascino M. D. Pei J. John A. Jahreis A. (2018). Long-Term Safety of Rituximab in Rheumatoid Arthritis: analysis from the SUNSTONE Registry. Arthritis Care Res. 71 (8), 993–1003. 10.1002/acr.23781 PMC6806017 30295434

[B67] Wollenhaupt J. Albrecht K. Krüger K. Müller-Ladner U. (2013). The new 2012 German recommendations for treating rheumatoid arthritis: differences compared to the European standpoint. Z. Rheumatol. 72 (1), 6–9. 10.1007/s00393-012-1093-6 23392597 PMC3567332

[B68] Yazici Y. (last update Jun 2018) Rheumatoid arthritis. BMJ Best Practice. Available at: bestpractice.bmj.com (Accessed 04/10/2019).

[B69] Yu Y. M. Lee E. Koo B. S. Jeong K. H. Choi K. H. Kang L. K. (2016). Predictive factors of spontaneous reporting of adverse drug reactions among community pharmacists. PloS One 11 (5), e0155517. 10.1371/journal.pone.0155517 27192159 PMC4871451

[B70] Zemkova M. Jebavy L. Kotlarova J. Vlcek J. Meyboom R. H. (2007). The spectrum and types of adverse side effects to biological immune modulators: a proposal for new classification. Folia Boil. 53 (4), 146–155. 10.14712/fb2007053040146 17706021

[B71] Zeltser R. Valle L. Tanck C. Holyst M. M. Ritchlin C. Gaspari A. A. (2001). Clinical, histological, and immunophenotypic characteristics of injection site reactions associated with etanercept: a recombinant tumor necrosis factor alpha receptor: Fc fusion protein. Arch. Dermatol. 137 (7), 893–899. 10-1001/pubs.ArchDermatol 11453808

[B72] Zhu J. Stuver S. O. Epstein A. M. Schneider E. C. Weissman J. S. Weingart S. N. (2011). Can we rely on patients’ reports of adverse events? Med. Care 49 (10), 948–955. 10.1097/MLR.0b013e31822047a8. 21642876

